# Argonaute 1 contributes to the transcriptional silencing of HIV-1

**DOI:** 10.1016/j.jbc.2025.110612

**Published:** 2025-08-19

**Authors:** Sophie Goudey, Melissa Ait Said, Juliette Desforges, Sébastien Marie, Marina Morel, Clarisse Berlioz-Torrent, Florence Margottin-Goguet, Stéphane Emiliani, Roy Matkovic, Sarah Gallois-Montbrun

**Affiliations:** Université Paris Cité, Institut Cochin, INSERM, CNRS, Paris, France

**Keywords:** HIV latency, argonaute 1, nuclear argonaute function, HIV transcriptional repression, miRNA-independent transcriptional control

## Abstract

HIV-1 latency remains a major barrier to viral eradication, and the mechanisms underlying the maintenance of proviral transcriptional silencing are not yet fully understood. Argonaute (Ago) proteins are well known for their roles in post-transcriptional gene silencing through microRNA-mediated pathways, but their involvement in transcriptional regulation, particularly in the context of HIV-1 infection, remains poorly characterized. Here, we demonstrate that Ago1 represses HIV-1 promoter activity across diverse latency models, independently of microRNA biogenesis pathways. Knockdown of Ago1, but not Ago2 or Dicer, significantly increased expression of latent proviruses in J-Lat cells, correlating with elevated nascent HIV-1 transcripts and reduced deposition of the repressive H3K9me3 mark at the LTR promoter. Single-molecule RNA FISH coupled with immunofluorescence revealed that Ago1 can accumulate at HIV-1 transcription sites upon activation, and biochemical analyses showed that Ago1 associates with chromatin and interacts with both initiating and elongating forms of RNA polymerase II. Notably, RNA was required for Ago1 chromatin recruitment but was dispensable for its interaction with RNA polymerase II. Using targeted HIV-1 promoter mutants, we further showed that Ago1-mediated repression is independent of key *cis*-regulatory elements, including the TAR stem-loop, the splice donor site SD1, and the polyadenylation signal. Collectively, our findings uncover a noncanonical role of Ago1 in HIV-1 transcriptional repression, further expanding its functional repertoire beyond RNA interference and providing new insights into the mechanisms maintaining HIV-1 latency, with possible implications for strategies aimed at reversing proviral silencing.

HIV persistence in latent reservoirs remains a major barrier to achieving a cure, as these reservoirs are highly heterogeneous in their location, provirus integrity, and replication potential. While defective proviruses dominate these reservoirs, intact integrated proviruses with varying reactivation dynamics are also present (1). Notably, even in individuals undergoing antiretroviral therapy, integrated proviruses are not uniformly transcriptionally silent. Instead, their transcriptional status is governed by a complex interplay of epigenetic, transcriptional, and post-transcriptional mechanisms acting at the proviral integration sites (2, 3). This heterogeneity in HIV latency poses a formidable challenge to developing therapeutic strategies aimed at eradicating the virus. Understanding the molecular factors that regulate proviral expression, particularly under latent conditions, is thus critical for informing cure strategies.

Several studies suggested that components of the miRNA pathway may influence HIV latency. Early findings proposed that miRNAs could directly target viral transcripts or indirectly modulate cellular factors involved in HIV transcription (4, 5, 6, 7). However, the direct involvement of miRNAs in regulating HIV-1 promoter activity during latency remained unclear (8, 9, 10). Interestingly, the microprocessor complex, composed of the RNase III enzyme Drosha and the dsRNA-binding protein DGCR8, has been directly implicated in basal HIV-1 promoter activity regulation. While these proteins are primarily known for their role in canonical pre-miRNA processing, Wagschal *et al.* (11) used HeLa LTR-Luciferase cells, a reporter model of HIV promoter transcriptional activity, to demonstrate that the microprocessor can activate HIV-1 transcription through mechanisms independent of miRNA biogenesis.

Downstream of Drosha and DGCR8, Argonaute (Ago) proteins are central players in the miRNA pathway, forming the core of the microRNA-induced silencing complex (12, 13). Although well known for their canonical role in miRNA-mediated translational regulation, Ago proteins also play broader roles in regulating cellular gene expression (14). Notably, the nuclear functions of Ago proteins have been extensively studied in fission yeast, where they are central to transcriptional gene silencing (TGS) (15). Similarly, in plants, Ago proteins are integral to ribonucleoprotein complexes that drive histone and DNA methylation (16).

In humans, two of the four Ago proteins, Ago1 and Ago2, have been implicated in a range of nuclear processes, including TGS and gene activation (17, 18, 19), alternative splicing (20, 21, 22, 23), and DNA repair (24, 25, 26). In particular, the nuclear functions of Ago1 have been described in several contexts, including its interaction with RNA polymerase II (RNAPII), its recruitment to active promoters and enhancers, and its role in alternative splicing, chromatin dynamics, and gene expression. These functions highlight its involvement in both canonical RNAi–related pathways and noncanonical transcriptional regulatory roles (14, 19, 22, 27, 28, 29, 30, 31, 32, 33).

In the context of HIV-1, we previously showed that Ago1 and Ago2 proteins bind to HIV RNA and are specifically recruited to splice donor sites. This recruitment regulates the alternative splicing of HIV-1 RNA, thereby influencing the viral transcriptome, and this, independently of miRNAs (34). However, the role of Ago proteins in modulating HIV expression under repressive conditions, such as latency, remains largely unexplored. To address this, we investigated the specific contribution of Ago proteins to HIV transcription using multiple latency models. Our findings reveal that Ago1 acts as a transcriptional regulator of the HIV-1 LTR promoter across different integration contexts and that its effects are dependent neither on miRNA-related mechanisms nor on specific *cis*-regulatory elements, such as TAR, SD1, or the polyadenylation signal (PAS). Overall, this study provides further evidence for a noncanonical role of Ago1 in transcriptional regulation and offers new insights into the regulation of latent HIV transcription. Our findings expand the established functional repertoire of Ago1 beyond RNA processing and highlight its potential as a therapeutic target for HIV-1 latency reversal strategies.

## Result

### Ago1 contributes to proviral repression in J-Lat models of HIV latency independently of the miRNA pathway

To address whether Ago proteins play a role in the regulation of HIV-1 expression during latency, we first evaluated the impact of their depletion in J-Lat models, which are Jurkat lymphoid cell lines containing a single provirus integrated at a well-characterized site (35). In J-Lat cells, the HIV-1 promoter is maintained at a repressed state, which can be reversed upon stimulation. Thus, this model provides a controlled system to investigate specific pathways involved in proviral repression.

**Figure 1:**
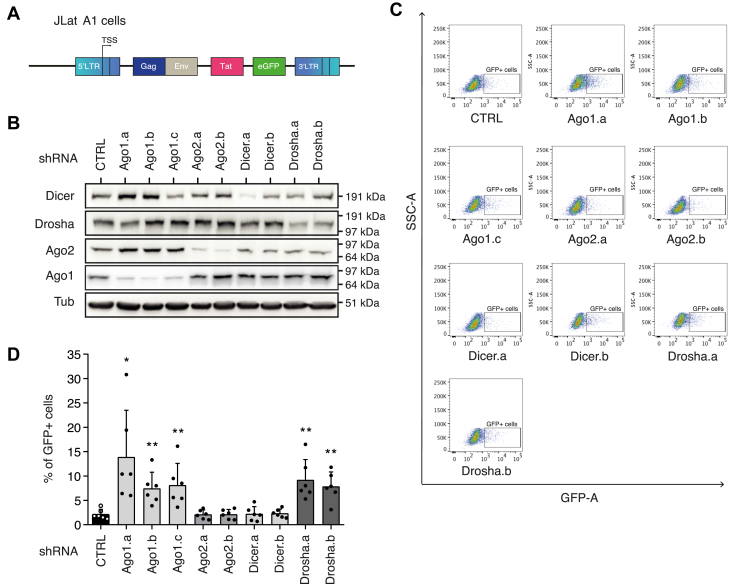
**Argonaute 1 downregulation enhances HIV-1 expression in J-Lat A1 model of viral latency, independently of the microRNA pathway**. J-Lat A1 cells were transduced with lentiviral vectors expressing either a control (CTRL) shRNA or shRNAs targeting components of the miRNA pathway. Seven days post-transduction, cells were harvested. *A,* schematic representation of the J-Lat A1 genome. *B,* knockdown efficiency in shRNA-transduced J-Lat A1 cells was monitored by immunoblotting using the indicated antibodies. A representative image is shown. *C,* flow cytometry analysis of GFP+ cells following transduction of J-Lat A1 cells with the indicated shRNAs. A representative experiment is shown. *D,* proviral expression, assessed by the percentage of GFP+ J-Lat A1 cells after transduction with shRNA CTRL or shRNAs targeting components of the miRNA pathway. Data represent mean ± SD (n = 6 independent experiments, with individual data points shown). *p* Values were calculated using unpaired two-tailed *t* test (∗*p* < 0.05; ∗∗*p* < 0.01).

Using short hairpin RNA (shRNAs), the expression of Ago1, Ago2, as well as Dicer and Drosha was first depleted in J-Lat A1 cells, which contain a copy of the LTR-Tat-GFP-LTR HIV minigenome (Fig. 1*A*). Protein knockdown (KD) was monitored by Western blot (Fig. 1*B*), and HIV-1 expression was monitored by measuring the percentage of GFP-expressing cells (Fig. 1*C*). Downregulation of Ago1 expression through transduction with three different shRNAs led to a 3.5- to 5.8-fold increase in the percentage of GFP-expressing cells (Fig. 1*D*).

Importantly, Ago2 downregulation by two different shRNAs did not lead to reactivation of HIV provirus expression, indicating that this role is specific to Ago1. Furthermore, KD of Dicer had no impact on the expression of HIV-1 provirus, suggesting that Ago1-mediated repression operates independently of miRNAs processed by Dicer. Nevertheless, we observed a significant increase in the percentage of GFP-expressing cells upon Drosha KD using two different shRNAs (Fig. 1*D*). This indicates that Drosha represses HIV-1 expression and confirms earlier findings obtained by Wagschal *et al.* (11) in a HeLa LTR-Luciferase reporter model. We further confirmed that only Ago1 KD leads to a 1.6- to 3-fold increase in the percentage of GFP-expressing cells in J-Lat 10.6 cells, a second model of HIV latency (Fig. S1), indicating that Ago1 depletion derepresses the expression of a nearly complete HIV-1 latent provirus.

Overall, these findings demonstrate that Ago1 plays a repressive role on the expression of HIV-1 provirus in lymphoid cells, independently of the miRNA pathway.

### Ago1 contributes to the transcriptional repression of the integrated latent provirus

**Figure 2:**
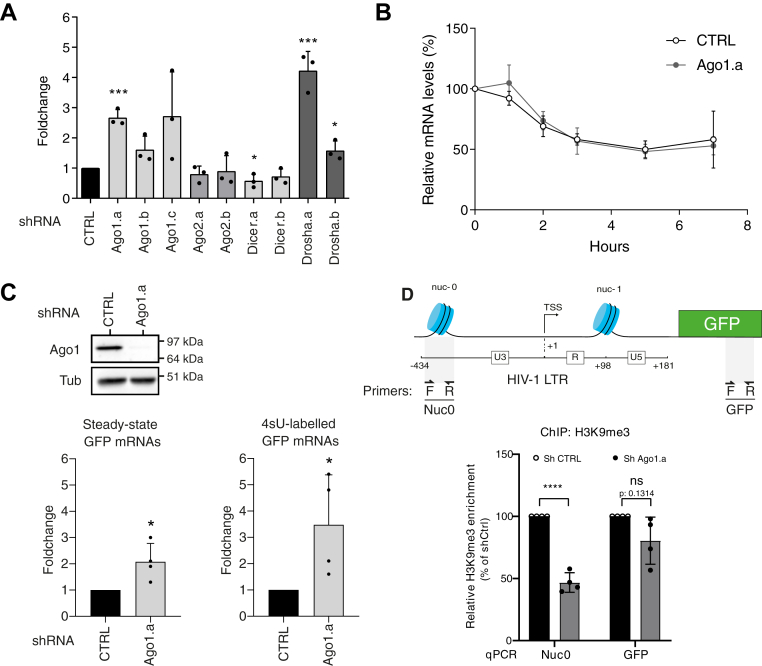
**Argonaute 1 (Ago1) downregulation relieves transcriptional repression at HIV-1 promoter.***A,* the steady-state level of HIV promoter–derived RNA was quantified by RT–qPCR in J-Lat A1 cells, 7 days post-transduction with either shRNA control (CTRL) or shRNAs targeting components of the miRNA pathway, as described in Figure 1. Data were normalized to shRNA CTRL and are presented as mean ± SD (n = 3 independent experiments, with individual data points shown). *p* Values were calculated using unpaired two-tailed *t* test (∗*p* < 0.05; ∗∗∗*p* < 0.001). *B,* the level of HIV promoter–derived RNA was quantified by RT–qPCR in J-Lat A1 cells transduced with shRNA CTRL or shRNA Ago1.a and treated with actinomycin D at the indicated time points (0, 1, 2, 3, 5, and 7 h). Data were normalized to the RNA level at *t* = 0 h, which was arbitrarily set to 100% within each condition. Data represent mean ± SD (n = 4 independent experiments). *C,* J-Lat A1 cells transduced with either shRNA CTRL or shRNA Ago1.a were incubated for 5 min with 4sU. 4sU-labeled RNA was subsequently isolated, and HIV promoter–derived RNA was quantified by RT–qPCR. Levels of LTR-driven GFP RNA levels were normalized to the levels of three housekeeping genes (*7SK*, *PIG-B*, and *KDSR*). Data represent mean ± SD (n = 3 independent experiments, with individual data points shown). *p* Values were calculated using a one-sample *t* test (∗*p* < 0.05). *D,* H3K9me3 enrichment at the HIV-1 promoter region (Nuc-0) and the GFP coding sequence in J-Lat A1 cells were assessed by ChIP–qPCR. Cells were transduced with either shRNA CTRL or shRNA Ago1.a. Signals were normalized to total H3 levels and expressed relative to the shRNA CTRL condition, arbitrarily set to 100%. The locations of the qPCR primers used for amplification of Nuc0 and GFP are indicated. Bar graphs represent the mean ± SD (n = 4 independent experiments, with individual data points shown). Statistical significance between shCTRL and shAgo1.a conditions was assessed using a Welch’s unequal variance two-tailed *t* test (∗*p* < 0.05). ChIP, chromatin immunoprecipitation; qPCR, quantitative PCR; 4sU, 4-thiouridine.

To elucidate the mechanisms by which Ago1 is involved in the repression of the integrated latent provirus, we examined the impact of Ago1 KD on the abundance of the LTR-driven GFP mRNA in the J-Lat A1 model using RT–quantitative PCR (qPCR). In correlation with the previously observed increase in the percentage of GFP-positive cells (Figs. 1*D*), Figure 2*A* revealed a significant increase (1.6-fold–2.7-fold) in steady-state GFP RNA levels upon Ago1 KD. This effect was specific to Ago1, as no similar increase was observed following KD of Ago2 or Dicer. As expected, KD of Drosha also resulted in an increase in GFP RNA levels, further correlating with the increase in GFP-positive cells. To determine whether this increase in LTR-driven mRNA abundance was due to changes in transcription rate, RNA stability, or both, we first evaluated the stability of GFP mRNA in J-Lat A1 cells following Ago1 KD. Cells were transduced with either control (CTRL) or Ago1-targeting shRNA (Ago1.a) and treated with actinomycin D for up to 7 h to inhibit transcription. As shown in Figure 2*B*, quantification of LTR-driven GFP mRNA levels revealed no significant difference between CTRL and Ago1 KD cells over this period, indicating that Ago1 does not affect the stability of LTR-driven transcripts.

To investigate whether Ago1 modulates transcriptional activity, we measured the levels of 4-thiouridine (4sU)-labeled newly synthesized RNAs. Cells were exposed to a 5-min pulse with 4sU, after which newly synthesized RNA was isolated and quantified *via* RT–qPCR. Figure 2*C* reveals a 3.3-fold increase in nascent GFP RNA levels upon Ago1 KD, consistent with the observed increase in steady-state GFP mRNA abundance. These findings indicate that Ago1 represses HIV-1 LTR expression at the transcriptional level.

Finally, we explored the potential impact of Ago1 KD in epigenetic regulation at the LTR promoter by analyzing the deposition of the repressive H3K9me3 histone mark *via* chromatin immunoprecipitation (ChIP). As shown in Figure 2*D*, Ago1 KD correlated with a twofold reduction in the H3K9me3 mark at the LTR promoter upstream of the transcription start site in the Nuc0 region. However, no significant reduction in H3K9me3 levels was observed at the GFP coding sequence, suggesting that Ago1 primarily exerts its effect at the promoter level. Taken together, our data demonstrate that Ago1 contributes to the transcriptional silencing and regulates H3K9me3 levels, a hallmark of heterochromatin, at the HIV-1 promoter.

### Ago1 associates with chromatin and interacts with RNAPII

**Figure 3:**
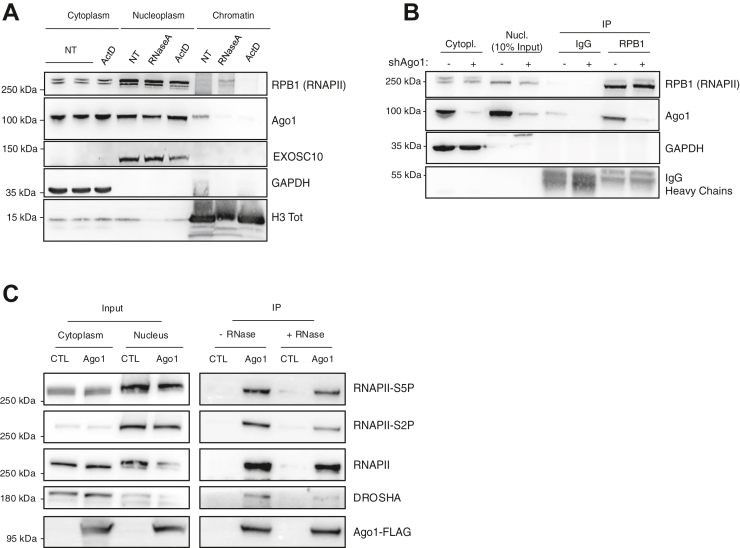
**Argonaute 1 (Ago1) interacts with initiating and elongating RNAPII in cells**. *A,* Ago1 is recruited to the chromatin fraction in an RNA-dependent manner. Cytoplasmic, nucleoplasmic, and chromatin-associated protein extracts were prepared from HeLa cells either untreated (NT) or treated with actinomycin D (ActD) at a 10 μg/ml final concentration to block transcription. Untreated cells were further either mock-treated or incubated with RNase A. Cytoplasmic, nucleoplasmic, and chromatin fractions were loaded on SDS-PAGE, and Ago1 and RNAPII (RPB1) were visualized using indicated antibodies. For each condition, three lanes are shown: NT (no ActD, no RNase), RNase A (no ActD, treated with RNase A), and ActD (ActD treated, no RNase A). GAPDH, EXOSC10, and histone H3 (H3 Tot) serve as markers for the cytoplasmic, nucleoplasmic, and chromatin fractions, respectively (n = 3 independent experiment, a representative image is shown). *B,* Ago1 interacts with endogenous RNAPII in nuclei of HeLa cells. Endogenous RNAPII was immunoprecipitated from nuclear extracts using an antibody targeting RPB1, the largest subunit of RNAPII. shAgo1-mediated Ago1 downregulation (6 days post shRNA transduction) served as a negative control for the interaction. GAPDH was used as additional negative and fractionation control. A representative image from n = 3 independent experiments is shown. *C,* reverse coimmunoprecipitation experiments were performed in HEK-293T cells either overexpressing, or not, Ago1-FLAG-HA, using an anti-FLAG antibody, in the absence (−) or presence (+) of RNase treatment. Ser5-phosphorylated (RNAPII-S5P) and Ser2-phosphorylated (RNAPII-S2P) forms of RNAPII serve as markers of the initiating and elongating transcriptional complexes, respectively. A representative result from n = 3 independent experiments is shown. HEK-293T, human embryonic kidney 293T cell line; RNAPII, RNA polymerase II.

While miRNAs are critical for Ago-mediated heterochromatin formation in fission yeast, their role or those of other noncoding RNAs in chromatin organization and transcriptional regulation in animal cells is unclear. Dicer inhibition in J-Lat A1 and J-Lat 10.6 cells did not increase LTR-driven GFP mRNA levels (Fig. 2*A*), suggesting that miRNAs are not involved in the process. Nevertheless, other RNAs may be involved. Previous studies have suggested that Ago1 contributes to transcriptional regulation and chromatin dynamics in mammalian cells by associating with promoters and/or enhancers and by interacting with RNAPII (22, 29, 31, 33, 36). We first confirmed by subcellular fractionation experiments in HeLa cells that Ago1 is present in both the nucleoplasmic and chromatin fractions (29, 31) (Fig. 3*A*). To assess the specificity of the fractionation process, we used GAPDH as a cytoplasmic marker, histone H3 as a marker for chromatin fractionation, and EXOSC10 as a marker for the nucleoplasmic fraction. Of note, under basal conditions, RNAPII was barely detectable in the chromatin fraction but became clearly enriched after RNase treatment. However, RNase treatment of the nuclear fraction significantly reduced Ago1 presence in the chromatin fraction, suggesting that RNA is necessary for Ago1 recruitment or for its stabilization on chromatin. Furthermore, blocking transcription and nascent RNA synthesis with actinomycin D prevented Ago1 association with chromatin (Fig. 3*A*). This suggests that chromatin-associated RNAs or nascent RNAs recruit Ago1 to chromatin. Coimmunoprecipitation (co-IP) of the largest subunit of endogenous RNAPII (RBP1) from the nuclear fraction of HeLa cells consistently revealed a robust interaction with Ago1 (Fig. 3*B*). Moreover, reciprocal IP experiments using Ago1-FLAG overexpressed in human embryonic kidney 293T (HEK-293T) cells further revealed that Ago1 associates with both Ser2- and Ser5-phosphorylated RNAPII, indicating interactions with both the initiating and elongating forms of RNAPII. Consistent with Huang *et al.* (29), our results further indicate that while the interaction between Ago1 and Drosha is predominantly RNA dependent, the association between Ago1 and RNAPII is mostly resistant to RNase treatment, suggesting an RNA-independent interaction (Fig. 3*C*). Together, these findings suggest that while RNA molecules facilitate Ago1 recruitment to chromatin, the interaction between Ago1 and RNAPII is mostly independent of RNA.

### Ago1 associates with HIV-1 promoter and regulates its transcriptional activity

**Figure 4:**
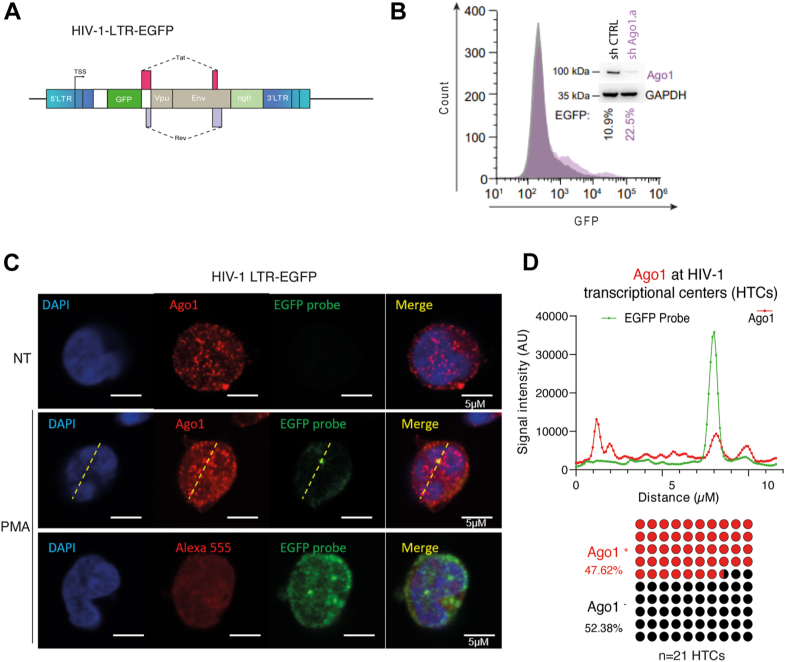
**Argonaute 1 (Ago1) can be recruited to HIV-1 transcription sites.***A,* schematic representation of the HIV-1-LTR-EGFP genome used to generate the HIV-1 polyclonal latent CD4+ Jurkat cell line. *B,* the Jurkat polyclonal latent cells were further transduced with either control shRNA or shRNA targeting Ago1, and EGFP expression was monitored by flow cytometry. A representative image of n = 2 experiments is shown. *C,* Jurkat cells harboring latent HIV-1 LTR-EGFP proviruses were *left* untreated (*top panels*) or treated with PMA (10 ng/ml, *middle and bottom panels*). Unspliced LTR-driven RNAs were detected by single-molecule RNA FISH using EGFP-specific probes (*green*), whereas endogenous Ago1 was visualized by immunofluorescence (*red*). In control experiments (*bottom panels)*, PMA-treated cells were stained with EGFP probes and secondary antibody only (Alexa 555) to validate the specificity of the anti-Ago1 primary antibody (a representative image of n = 2 independent experiment is shown). Nuclei were counterstained with DAPI (*blue*). Scale bars represent 5 μm. *D,* quantification of fluorescence signal intensities along the *yellow line* shown in (*C*), illustrating the method used to assess the colocalization between endogenous Ago1 and HIV-1 transcriptional centers (HTCs) identified by smFISH. *Bottom panel,* quantification of Ago1 signal examined over 21 HTCs (n = 2 independent experiments). DAPI, 4′,6-diamidino-2-phenylindole; EGFP, enhanced GFP; PMA, phorbol 12-myristate 13-acetate.

To investigate whether Ago1 is recruited to the HIV-1 promoter, we then examined its distribution in a polyclonal Jurkat cell model of HIV-1 latency, containing HIV-1-LTR-enhanced GFP (EGFP) proviruses at different genomic loci (37). The proviruses contain complete LTRs, express Tat, and Rev, but possess an *egfp* cassette instead of *gag*, *pol*, *vif*, *vpr*, and harbor a frameshift mutation in *env*, and an *ngfr* reporter gene in the place of *nef* (38) (Fig. 4*A*). As expected, knocking down Ago1 expression from these cells led to a twofold increase of EGFP expression (10.9–22.5%) (Fig. 4*B*). To assess whether Ago1 can be recruited to the integrated provirus at its locus, we then employed single-molecule RNA FISH coupled to immunofluorescence (IF) to label HIV-1 LTR–driven transcript. As previously described, endogenous Ago1 labeling showed a nuclear distribution with an accumulation in nuclear foci (29) (Fig. 4*C*). Upon phorbol 12-myristate 13-acetate treatment (10 ng mL^−1^), labeling the HIV-1 LTR–driven transcripts enabled detection of the HIV-1 transcription center (provirus locus). We observed that 47% of viral genomic RNA spots, as detected by single-molecule RNA FISH, colocalize with nuclear Ago1 foci (Fig. 4, *C* and *D*). This enrichment was consistent across almost half of the cells, aligning with position-effect variegation, and suggests that Ago1 is recruited to HIV-1 active transcriptional sites.

### Ago1 represses HIV-1 promoter activity independently of TAR, SD1, and PAS elements

**Figure 5:**
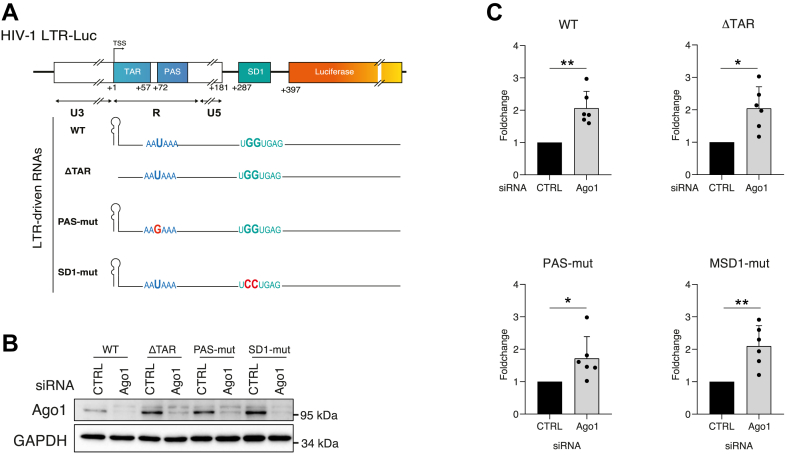
**Argonaute 1 (Ago1) participates in the HIV-1 promoter repression in HeLa LTR-Luc model**. *A,* schematic representation of the HIV-1 LTR-Luc provirus integrated into HeLa cells, along with the point mutations introduced in the TAR stem-loop (ΔTAR), the polyadenylation signal (PAS-mut), and the splice donor site D1 (SD1-mut). *B,* the knockdown efficiency in HeLa LTR-Luc cells transfected with either siRNA CTRL or siRNA targeting Ago1 was monitored by immunoblotting using the indicated antibodies. *C,* Luc RNA levels were quantified by RT–quantitative PCR (qPCR) in either WT HeLa HIV-1 LTR-Luc cells or carrying indicated mutations (ΔTAR, PAS-mut, and SD1-mut) following transfection with either siRNA CTRL or siRNA targeting Ago1. Data represent mean ± SD (n = 6 independent experiments, with individual data points shown). *p* Values were calculated using a one-sample *t* tests (∗*p* < 0.05; ∗∗*p* < 0.01; and ∗∗∗*p* < 0.001).

Our data indicate that Ago1 downregulation increases HIV-1 promoter activity across different integration contexts, suggesting that Ago1 influences viral promoter activity independently of the cellular context. To investigate whether specific features of the HIV-1 promoter contribute to this regulation, we employed a previously established HIV-1 luciferase reporter system (HIV-1 LTR-Luc) and corresponding promoter mutants (Fig. 5*A*). These mutations affect three key regions: the TAR element, which is essential for HIV-1 transcriptional activation and may also contribute to the production of TAR-derived small RNAs (39, 40, 41); the SD1 splice donor site, known to recruit Ago proteins (34); and the PAS, which is involved in transcription termination at the 3′LTR, and must be inactivated at the 5′LTR through the interaction of U1 snRNP with SD1 during active HIV-1 transcription (42). To minimize integration site effects, WT and mutated LTR constructs were inserted at the same locus in HeLa Flp-In cells (43). Ago1 depletion by siRNA resulted in a similar increase of Luc expression in all constructs, indicating that Ago1-mediated repression is independent of *cis*-acting elements, such as TAR, SD1, and PAS (Fig. 5, *B* and *C*).

### Ago1 KD enhances HIV-1 expression in infected Jurkat cells

**Figure 6:**
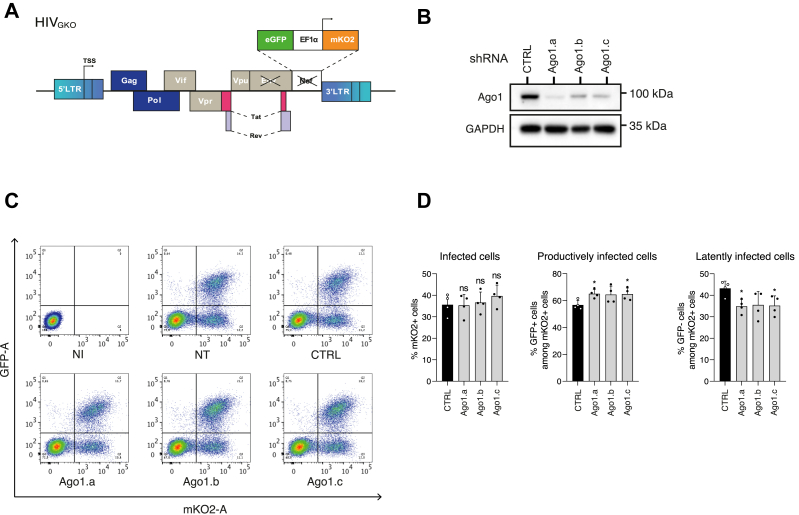
**Argonaute 1 (Ago1) represses the expression of HIV-1_GKO_ provirus in CD4+ Jurkat cells.** Following infection with HIV_GKO_ virus for 24 h, CD4+ Jurkat cells were transduced with lentiviral vectors expressing either control (CTRL) shRNA or three different shRNAs targeting Ago1. Five days post-transduction, cells were harvested. *A,* schematic representation of the HIV_GKO_ genome. *B,* Ago1 knockdown efficiency after shRNA transduction of Jurkat cells and infection with HIV_GKO_ was monitored by immunoblotting using Ago1 antibody (representative image of n = 3 independent experiments). *C,* flow cytometry analysis of GFP+ and mKO2+ cells following infection with HIV_GKO_. A representative experiment is shown. *D,* the relative level of productively infected Jurkat cells (mKO2+, GFP+) and latently infected Jurkat cells (mKO2+, GFP−) was monitored after transduction with shRNA CTRL or three different shRNA targeting Ago1. Data represent mean ± SD (n = 3 independent experiments, with individual data points shown). *p* Values were calculated using two-tailed unpaired *t* test (∗*p* < 0.05; ∗∗*p* < 0.01).

While J-Lat models are useful for dissecting mechanisms at the level of a single integration site, we aimed to confirm our findings in a dynamic and relevant context. To confirm whether Ago1 participates in regulating HIV proviral expression, we utilized the dual-labeled HIV_GKO_ reporter virus, which more closely mimics natural infection by allowing the study of HIV expression across multiple integration sites (44). This system features a quasi-intact HIV-1 89.6 genome in which the *nef* gene is replaced by *GFP* under the control of the viral promoter, whereas a monomeric Kusabira orange 2 **(**mKO2) expression is driven by the EF1α promoter (Fig. 6*A*). This dual-reporter configuration enables monitoring of the global percentage of infected cells (mKO2+) and distinguishing between cells with active proviral expression (mKO2+, GFP+) and those with low or absent viral promoter activity (mKO2+, GFP-). Jurkat cells, a CD4+ T cell line, were first infected with HIV_GKO_ for 24 h. Cells were then subjected to Ago1 KD using three distinct shRNAs. Five days post-transduction, the KD efficiency was verified by Western blotting (Fig. 6*B*), and the proportions of GFP+ and mKO2+ cells were quantified by flow cytometry (Fig. 6*C*). Overall, Ago1 KD had no significant effect on the global percentage of mKO2-expressing cells, indicating that Ago1 does not impact the proportion of cells containing an integrated provirus (Fig. 6*D*, *left panel*). However, Ago1 KD led to a redistribution in the infected population: the proportion of productively infected cells (mKO2+, GFP+) significantly increased from 56 up to 62%, whereas the proportion of latently infected cells (mKO2+, GFP-) dropped from 43 to 38% (Fig. 6, *C* and *D*). These findings reinforce the role of Ago1 in limiting expression of a subset of integrated proviruses, further supporting its contribution to HIV transcriptional regulation in the context of infection.

## Discussion

**Figure 7:**
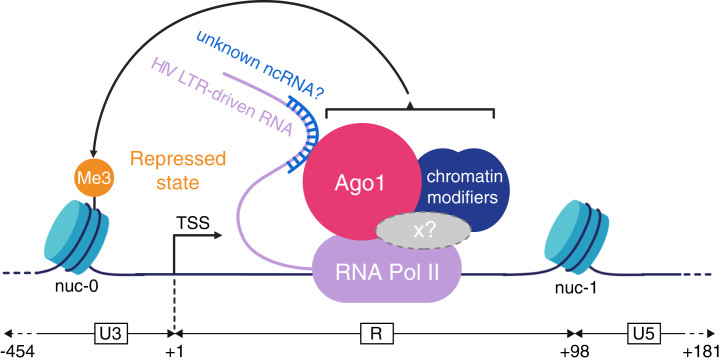
**Model of the role of Argonaute 1 (Ago1) in transcriptional repression of the HIV-1 promoter.** Ago1 is recruited to the HIV-1 promoter likely through RNA-dependent interactions, potentially involving LTR-driven nascent transcripts, possibly noncoding RNAs, but independently of miRNAs. Once associated with chromatin and RNA polymerase II (RNAPII), Ago1 may contribute to transcriptional repression either directly or by stabilizing a repressive chromatin environment at the promoter. This repressive function appears specific to Ago1 and does involve neither canonical miRNA pathway components nor the microprocessor or HUSH complexes. While mechanistically distinct from the RITS complex in *Saccharomyces pombe*, whose closest functional counterpart in human cells is the HUSH complex, this mechanism may reflect an evolutionary echo of RNA-guided transcriptional silencing strategies observed in lower eukaryotes. Ago1 may then serve as a scaffold for chromatin-modifying machineries, facilitating heterochromatinization of the viral promoter and contributing to the HIV-1 transcriptional silencing. RITS, RNA-induced transcriptional silencing.

Ago1 has been previously shown to act as either a repressor or an activator of cellular gene transcription, underscoring its dual and context-dependent regulatory roles (27, 31, 32, 33, 45). In this study, we demonstrate that Ago1 KD consistently derepresses HIV-1 expression across different models of transcriptional repression of the viral promoter. This correlates with an increase in newly synthesized 4sU-labeled mRNA levels and a twofold reduction in the H3K9me3 repressive mark at the LTR promoter (Nuc0 region), highlighting its involvement as a transcriptional repressor of the HIV-1 promoter in the context of latency (Fig. 2, *C* and *D* and model in Fig. 7). Interestingly, the downregulation of Ago2 and Dicer, two key components of the miRNA pathway, did not induce HIV-1 promoter reactivation in our models (Figs. 1*D* and 2*A*). This observation strongly supports the notion that Ago1-mediated transcriptional repression of the viral promoter is independent of canonical miRNA activity. These findings are consistent with the results of Wagschal *et al.* (11), who showed that while microprocessor is significantly involved in HIV-1 promoter repression, the downregulation of Dicer or Ago2 had no effect on the viral promoter activity. However, in contrast to their study, we found that robust downregulation of Ago1, achieved using either siRNA or three different shRNAs across various models of transcriptional repression, reproducibly led to an increase of the HIV-1 promoter activity (Figure 1, Figure 2, Figure 4 and 5 and Fig. S1). A plausible explanation for this discrepancy lies in the efficiency of Ago1 KD. Interestingly, while Drosha and DGCR8 modulate HIV-1 promoter activity through interactions with the TAR stem-loop, a critical *cis*-regulatory motif within the LTR, we found that Ago1-mediated repression operates independently of TAR (Fig. 5*C*) (11). This highlights that Ago1 and the microprocessor complex regulate HIV-1 transcription through distinct, nonoverlapping mechanisms.

It was previously shown that transcriptional regulation of the HIV-1 promoter can be achieved *via* Ago2 and Ago1 when guided by synthetic siRNAs or shRNAs, leading to TGS through epigenetic modifications or recruitment of repressive complexes (45, 46, 47, 48, 49). However, our study reveals that in the absence of such guidance, Ago1, not Ago2, represses the HIV-1 promoter. Ago1 and Ago2 are structurally very similar, sharing around 85% sequence identity (50). Despite this similarity, a critical functional distinction lies in that Ago2 retains slicing activity, enabling cleavage of perfectly complementary target RNAs, whereas Ago1 lacks this capacity. Interestingly, parallels can be drawn with the Piwi-clade Ago, such as Piwi in *Drosophila*, where slicer-incompetent Piwi directs the deposition of H3K9me3 marks and represses RNAPII occupancy at the transposon loci. Yamagushi *et al.* (51) even suggested that a slicer activity could compromise its ability to mediate cotranscriptional silencing. Similarly, the decrease of H3K9me3 at the HIV-1 promoter upon Ago1 KD suggests a function in chromatin-based silencing, without the requirement of the slicer activity. It would be intriguing to investigate whether restoring slicing activity to Ago1 would compromise its transcriptional regulatory function or, conversely, whether abolishing slicing activity in Ago2 might reveal a latent role in transcriptional silencing (52). These experiments could provide further insight into the relationship between slicing activity and transcriptional regulation in the Ago family.

Importantly, in *Saccharomyces pombe*, Ago1 complexed to siRNAs associates with ChP1 and Tas3 to form the RNA-induced transcriptional silencing (RITS) machinery that targets nascent transcripts and induces gene silencing through H3K9me3 deposition and heterochromatin formation (15, 53). We and others have reported that the HUSH complex participates in the repression of HIV-1 LTR expression and shares functional and structural similarities with RITS (37, 54, 55, 56). Supporting an RITS-like role for Ago1 at the HIV-1 LTR, ChIP experiments revealed a reduction in H3K9me3 levels upstream of the transcription start site following Ago1 KD (Fig. 2*D*). This is consistent with previous observations showing that siRNAs targeting the U3 region promote Ago1 recruitment and H3K9me3 deposition (49). However, no significant change in H3K9me3 was detected over the downstream GFP region, where deposition depends on HUSH activity (55). This suggests that Ago1 negatively regulates HIV-1 expression through a HUSH-independent mechanism.

To further investigate the mechanism by which Ago1 regulates the HIV-1 viral promoter, we utilized TAR, SD1, and PAS mutants inserted at a single genomic locus in HeLa Flp-IN cells. Our results revealed that Ago1-mediated repression of LTR activity is independent of these key *cis*-regulatory elements, ruling out their direct involvement (Fig. 5). However, it is possible that Ago1 recognizes secondary RNA structures or interacts with other viral elements within LTR-driven nascent RNA to mediate transcriptional repression. Indeed, cell fractionation experiments confirmed that the recruitment or stabilization of Ago1 to chromatin is RNA dependent (Fig. 3*A*).

Consistent with its role in transcriptional regulation of cellular genes, we confirmed that Ago1 interacts with RNAPII and further showed that this interaction occurs with both the initiating and elongating forms of the RNAPII. Surprisingly, and contrary to shuaib *et al.* (31), but in agreement with Huang *et al.* (29), the interaction of Ago1 and RNAPII was independent of the presence of RNA (Fig. 3, *B* and *C*). Shuaib *et al.* (31) further suggested that Ago1 contributes to maintaining chromatin organization and, consequently, influences gene expression, supporting a broader role for Ago1 in transcriptional regulation and chromatin dynamics. Previous studies also showed that Ago1 is strongly enriched at transcriptionally active enhancers and promoters of cellular genes, possibly *via* interactions with RNA intermediates, such as enhancer RNAs (22, 31, 33, 36). Given that HIV-1 preferentially integrates into transcriptionally active regions of the genome, it is possible that Ago1 exploits this enhancer-related activity to interact with nearby regulatory elements at integration sites, thereby modulating LTR activity. While our data from J-Lat A1, J-Lat 10.6, and HeLa LTR-Luc cells and models with multiple integration sites (HIV-1-LTR-GFP and HIV_GKO_) indicate that Ago1 can repress LTR-driven transcription across diverse integration contexts, the modest increase in the proportion of actively infected HIV_GKO_ cells following Ago1 KD suggests that its repressive role may also be influenced by the chromatin environment at the integration site (Figs. 1, 2, 4, 5 and 6). As for the RITS complex in fission yeast, recruitment of Ago1 to the HIV-1 provirus may serve as a scaffold for chromatin-associated proteins or transcriptional repressors, establishing a repressive environment around the LTR (Fig. 7). While ChIP of Ago1 would be a valuable approach to precisely map its binding sites at the HIV-1 promoter, repeated attempts did not yield reproducible results. This may reflect the low abundance or dynamic turnover of Ago1 at the viral promoter or the limited accessibility of the epitope recognized by the available antibodies. Nevertheless, while we cannot entirely rule out the possibility that Ago1 depletion indirectly affects HIV promoter activation through the regulation of key transcription factors, our IF experiments revealed that Ago1 can specifically accumulate at active HIV-1 transcription sites (Fig. 4).

Overall, this study reveals a noncanonical role for Ago1 in repressing HIV-1 transcription during latency. Our findings show that Ago1 contributes to proviral silencing independently of miRNA pathways and known *cis*-regulatory elements. By associating with chromatin and RNAPII, Ago1 likely participates in maintaining a repressive chromatin environment at the viral promoter (Fig. 7). Future studies will be needed to further define the molecular mechanisms underlying Ago1 recruitment and activity, including its interactions with chromatin-associated factors and regulatory RNAs. A deeper understanding of these processes may offer new opportunities to target latent reservoirs and advance HIV-1 eradication strategies.

## Experimental procedures

### Cell lines

Jurkat lymphocyte line and Jurkat clones, J-Lat A1 and 10.6, were obtained from the National Institutes of Health HIV reagent program; HEK-293T cells (CRL-3216) were obtained from American Type Culture Collection. The Jurkat polyclonal latent HIV-1 EGFP cells were generated and described (37). All these Jurkat cells were cultured in RPMI1640 medium (Gibco Life Technologies) plus GlutaMAX. Adherent cells, HEK-293T, HeLa, and HeLa LTR-Luc cells with a single integrated copy of the *luciferase* gene under the CTRL of WT or mutated HIV-1 promoters described (43) were cultured in Dulbecco's modified Eagle's medium (Gibco, Life Technologies) plus GlutaMAX. All media were supplemented with 10% fetal calf serum (SVF, GibcoBRL) and 1% antibiotics–antimycotics (Gibco, 15240-062). All cells were cultured at 37 °C under 5% CO_2_. Cell lines were regularly tested for mycoplasma contamination.

### Production and transduction of pseudotyped viruses

The HIV_GKO_ provirus was a kind gift from Eric Verdin (44). For the production of pseudotyped viral stocks, 5 × 10^6^ HEK-293T cells were transfected with 4.5 μg HIV_GKO_ plasmid and 1.5 μg VSV-G protein vector using polyethylenimine (Polyscience). Cells were washed 12 h post-transfection, and viral stocks were collected 48 h post-transfection and filtered. Viral stock concentration was determined using HIV-1 p24GAG antigen quantification *via* ELISA (PerkinElmer; NEK050b001KT).

For Jurkat cell infection, 1 × 10^7^ cells were infected with 900 ng p24GAG VSV-G-pseudotyped HIV_GKO_ viral preparation for 2 h and then washed with PBS and cultured for 5 days before shRNA transduction.

### Gene silencing *via* siRNA transfection and shRNA transduction

For siRNA transfection, 2 × 10^5^ HeLa HIV LTR-Luc cells were transfected with 37.5 nM siRNA using Lipofectamine RNAiMAX (ThermoFisher Scientific) and harvested 72 h later.

For shRNA cloning, RNA oligonucleotides (Table S1) were annealed and inserted into the pLKO.1 lentiviral vector (Addgene #10878) according to the Addgene protocol (http://www.addgene.org/tools/protocols/plko/). Virus-like particles expressing the shRNAs were produced by transfecting 5 × 10^6^ HEK-293T cells with 3 μg pLKO.1 vector containing the specific shRNA sequence, 2.25 μg psPAX2 (Addgene #12260) and 0.75 μg VSV-G protein vector using polyethylenimine. Medium was replaced 12 h post-transfection, and viral stocks were collected 48 h post-transfection. Viral stock concentration was determined by HIV-1 p24GAG antigen quantification using ELISA (PerkinElmer, NEK050b001KT). For HIV_GKO_ infection, 1 × 10^7^ Jurkat cells were incubated with 900 ng p24GAG VSV-G-pseudotyped HIV_GKO_ viral preparation for 2 h at 37 °C, then washed with PBS, and cultured at a 10^6^ cells/ml density. Five days postinfection with HIV_GKO_, 2 × 10^6^ Jurkat cells were transduced with 300 μl of shRNA production by incubation for 3 h at 37 °C. Similarly, 2 × 10^6^ J-Lat A1 and J-Lat 10.6 cells were transduced with 300 μl of shRNA production. Twenty-four hours post-transduction, cells were selected with the antibiotic puromycin (Gibco) at a final concentration of 1 μg/ml and harvested and analyzed by flow cytometry and immunoblotting 7 days post-transduction.

### Flow cytometry analysis

About 2 × 10^6^ J-Lat cells or HIV_GKO_-infected Jurkat cells transduced with shRNA were analyzed on an LSR Fortessa flow cytometer (Beckton Dickinson) after live-dead (Invitrogen; L23105A) labeling and fixation with 4% Pierce formaldehyde (ThermoFisher Scientific; 28908). Data were analyzed with the FlowJo software (Beckton Dickinson).

### RNA extraction, reverse transcription, and qPCR

RNA was extracted from cells using an RNA extraction kit (Z6012; Promega). RNA quality and quantity were assessed, and 1 μg of RNA were treated with DNase to remove contaminating DNA (Turbo DNase; AM2239, Invitrogen). Reverse transcription was performed to generate complementary DNA (cDNA) using High-Capacity cDNA Reverse Transcription Kit with random primers (Applied Biosystems by Life Technologies; 4368813). cDNA samples were diluted 1:10, and 5 μl of each sample was loaded in duplicate into a 96-well plate preloaded with 15 μl of reaction mix. The reaction mix consisted of 10 μl of SYBR Green Master Mix (Bio-Rad), 1 μl of forward primer (10 μM), 1 μl of reverse primer (10 μM**)** (Table S1), and 3 μl of H_2_O. Plates were analyzed using the LightCycler 480 system (Roche) under the following cycling conditions: 95 °C for 5 min, followed by 40 to 45 cycles of 95 °C for 10 s, 58 to 60 °C for 10 to 30 s, and 72 °C for 10 to 30 s, with a final step at 40 to 65 °C for 30 s. Results were analyzed using the ΔΔCt method, with transcript levels normalized to *GAPDH* or *KDSR* Ct values.

### Immunoblotting

Cellular proteins were extracted using radioimmunoprecipitation assay lysis buffer (150 mM NaCl, 10 mM Tris [pH 8], 0.5% Triton, 1 mM EDTA, and 0.1% sodium deoxycholate) supplemented with cOmplete Protease Inhibitor Cocktail (Roche). After cell lysate clarification by centrifugation at 21,000*g* for 30 min, protein concentrations were determined using the Bradford Protein Assay (Bio-Rad). A total of 20 to 50 μg of protein was mixed with 2X Laemmli buffer (Sigma–Aldrich), heated at 70 to 95 °C for 5 min, and separated either on 10% SDS-PAGE acrylamide gels in Tris–glycine–SDS buffer (Euromedex) at 25 mA or on NuPage 4 to 12% Bis–Tris acrylamide gels (Life Technologies) in Mops–SDS buffer (Life Technologies) at 30 mA. Proteins were transferred to polyvinylidene fluoride membranes (0.45 μm; Immobilon) using wet transfer in Tris–glycine buffer with 20% methanol (Euromedex) for 2 h at 200 mA. Membranes were blocked in Tris-buffered saline with 0.1% Tween-20 and 5% milk or 5% bovine serum albumin for 30 min at room temperature, followed by overnight incubation at 4 °C with the specified primary antibodies: anti-Ago1 4B8 (SAB4200084; Sigma), anti-Ago1 D84G10 (5053; Cell Signaling), anti-Ago2 C34C6 (2897; Cell Signaling), anti-Dicer A-2 (sc-136981; Santa Cruz), anti-Drosha D28B1 (3364; Cell Signaling), anti-RNA Pol II F-12 (sc-55492; Santa Cruz), anti-RNA Pol II phospho-Ser2 E1Z3G (13499; Cell Signaling), anti-RNA Pol II phospho-Ser5 (ab5131; Abcam), anti-GAPDH (sc-47724; Santa Cruz), and anti-α-Tubulin (T9026; Sigma–Aldrich). Horseradish peroxidase–conjugated anti-mouse (P0260), anti-rat (P0450), or anti-rabbit (P0217) IgG secondary antibodies (Dako) were applied for 1 h at room temperature. Signals were detected using either Amersham ECL Select Western-blotting detection reagent (Merck) or Immobilon Crescendo or Forte Western horseradish peroxidase substrates (Merck). Images were acquired using a Fusion FX imaging system (Vilber).

### Assessment of viral stability

Seven days after transduction with either CTRL shRNA or shRNA targeting Ago1, 3 × 10^6^ J-Lat A1 cells were treated with actinomycin D (5 μg/ml; A9415-5MG) for 0, 1, 2, 3, 5, and 7 h. Cells were lysed, and RNA was extracted using the ReliaPrep RNA extraction kit (ReliaPrep RNA Cell Miniprep System; REF Z6012). Total viral RNA levels were quantified by RT–qPCR (57) (Table S1).

### 4sU labeling of newly synthesized HIV-1 RNAs

The metabolic labeling of RNAs with 4sU was conducted following the optimized protocol (58). In brief, 10^7^ J-Lat A1 cells were incubated in 1 ml of RPMI medium containing 20 μM 4sU (Sigma–Aldrich; T4509) for a 5-min pulse at 37 °C in a 5% CO_2_ incubator. Following labeling, cells were centrifuged at 600*g* for 3 min at 4 °C, and the resulting cell pellets were lysed using 1 ml of Trizol reagent (Invitrogen; 15596018). A total of 100 μg of extracted RNA was biotinylated using NHS-Biotin EZ-Link (Thermo Scientific; 21341) and subsequently precipitated on Streptavidin T1 Dynabeads MyOne (Invitrogen; 65602). For analysis, 1 μg of input RNA was set aside prior to IP and later assessed by RT–qPCR alongside the eluted RNA samples. RT–qPCR analysis was performed (43).

### Cell fractionation and coimmunopurifications

Cytoplasmic, nucleoplasmic, and chromatin-bound protein fractions were isolated using a sequential extraction protocol. All buffers were prepared with nuclease-free water and supplemented with protease inhibitors (cOmplete; Roche) immediately before use. Cells were treated or not with actinomycin D (A4262-5MG; Sigma–Aldrich) at a 10 μg/ml final concentration for 30 min. They were then collected by centrifugation (300*g*, 5 min, 4 °C) and resuspended in ice-cold hypotonic lysis buffer (10 mM Tris–HCl [pH 7.5], 10 mM NaCl, 3 mM MgCl_2_, 0.3% NP-40, and 10% glycerol) at a ratio of 1 ml per 10 million cells. After a 10-min incubation on ice, samples were briefly vortexed and centrifuged at 800*g* for 8 min at 4 °C. The supernatant containing the cytoplasmic fraction was collected and stored on ice. The nuclear pellet was then treated or not with RNase A (70856; Millipore) with 16 U for 15 min at 37 °C to assess the distribution of Ago1 upon nuclear RNA removal. The nuclear pellet was washed four times with hypotonic lysis buffer (200*g*, 2 min, 4 °C) and then resuspended in 0.5 ml of modified Wuarin–Schibler buffer (10 mM Tris–HCl [pH 7.0], 4 mM EDTA, 0.3 M NaCl, 1 M urea, and 1% NP-40) supplemented with protease inhibitors. Samples were vortexed and centrifuged at 1000*g* for 5 min at 4 °C. The supernatant, corresponding to the nucleoplasmic fraction, was transferred to a clean tube and stored on ice. The chromatin-bound fraction was obtained by washing the chromatin pellet twice with ice-cold modified Wuarin–Schibler buffer (vortexing followed by 5 min on ice and centrifugation at 500*g* for 3 min). Chromatin was then resuspended in 300 μl of nuclear lysis buffer (20 mM Tris–HCl [pH 7.5], 150 mM KCl, 3 mM MgCl_2_, 0.3% NP-40, and 10% glycerol), followed by sonication using a Bioruptor (Diagenode; 10 cycles, 30 s ON/30 s OFF, high setting). Sonicated samples were placed on ice immediately. All collected fractions (cytoplasmic, nucleoplasmic, and chromatin bound) were clarified by centrifugation at 32,000 rpm for 10 min at 4 °C and processed for downstream applications. Co-IP of endogenous RNAPII (RPB1) was performed using 1 mg of nuclear extracts (37). Briefly, cells were fractionated to isolate intact nuclei. Nuclei were then lysed in 600 μl of ice-cold SDS-free lysis buffer (50 mM Tris–HCl [pH 7.5], 150 mM NaCl, 2 mM EDTA, 10% glycerol, and 0.5% NP-40). Total protein concentration was determined, and 500 μg of protein was used per IP. The volume was adjusted to 500 μl with washing buffer. Five percent of each sample (25 μg) was retained as input. For RPB1 IP, 4 μg of the F12 antibody (sc-55492; Santa Cruz Biotechnology) or mouse irrelevant IgG (10283-1-AP; Proteintech) were incubated overnight at 4 °C with samples on a rotating wheel. Protein–antibody complexes were incubated for 30 min at room temperature with 25 μl of prewashed Protein G beads (Thermo Fisher Scientific) per IP. Beads were washed three times on a magnetic rack: twice with 1 ml and once with 500 μl cold washing buffer (50 mM Tris–HCl [pH 7.5], 150 mM NaCl). Beads were resuspended in 100 μl of washing buffer containing protease inhibitors, which was added to the beads, followed by 25 μl of 5× Laemmli sample buffer. Samples were mixed gently and boiled at 95 °C for 5 to 10 min. Tubes were then cooled on ice and briefly centrifuged. Supernatants containing immunoprecipitated proteins were subjected to SDS-PAGE and Western blot analysis.

For the reverse co-IP, HEK-293T cells (1.2 × 10^8^ per condition) were transfected with either the CTRL (FLAG-HA-pcDNA3.1; plasmid #52535) or the Ago1 overexpression plasmid (pIRESneo-FLAG/HA Ago1; plasmid #10820). Cells were harvested 48 h post-transfection and first resuspended in hypotonic buffer (10 mM Tris, pH 7.3; 10 mM KCl; 1.5 mM MgCl_2_) before centrifugation at 1000*g* for 5 min. After careful removal of the supernatant, cells were resuspended in hypotonic buffer, incubated on ice for 10 min, and passed through a Dounce homogenizer. After centrifugation at 2600*g* for 15 min at 4 °C, the resulting nuclear pellet was resuspended in low salt buffer (20 mM Tris, pH 7.3; 12.5% glycerol; 1.5 mM MgCl_2_; 0.2 mM EDTA; 0.2 mM PMSF; and 10 mM β-mercaptoethanol) and passed through a Dounce homogenizer, with addition of high salt buffer (20 mM Tris, pH 7.3; 12.5% glycerol; 1.5 mM MgCl_2_; 0.2 mM EDTA, pH 8; 0.2 mM PMSF; 10 mM β-mercaptoethanol; and 1.2 M KCl) during the homogenization process until reaching a concentration of 600 mM KCl. Nuclear extracts were then placed on a wheel at 4 °C for 30 min and later centrifuged at 1500*g* for 30 min. The IP was performed with 1 mg of nuclear extracts, all brought to the same volume in IP buffer (20 mM Tris, pH 7.3; 12.5% glycerol; 1.5 mM MgCl_2_; 0.2 mM EDTA, pH 8; 0.2 mM PMSF; 10 mM β-mercaptoethanol; 200 mM KCl; and 0.2% Triton). RNase treatment was performed on half of the samples by addition of 5 U of RNase A (Roche), followed by incubation at 30 °C for 10 min. Finally, samples were incubated overnight at 4 °C on a wheel after addition of 3 μg of ANTI-FLAG antibody (ANTI-FLAG M2 antibody, F3165; Sigma–Aldrich). After two washes in IP buffer, Dynabeads Protein G were added to the samples and incubated on a wheel at 4 °C for 4 h. To eluate, beads were resuspended in 4X Laemmli buffer and heated at 70 °C for 10 min.

### ChIP–qPCR

J-Lat A1 shCTRL and shAgo1.a cells were cultured in glutamine-supplemented RPMI medium at a cell density of 5 × 10^5^ cells per ml. ChIP experiments were performed using the HighCell# ChIP kit (Diagenode) according to the manufacturer’s instruction but with 10 rounds of sonication of 30 s on/30 s off with a Bioruptor Pico (Diagenode) (55). The following antibodies were used for ChIP: anti-H3Pan (C15310135; Diagenode), anti-H3K9me3 (C15410056; Diagenode), and anti- and the rabbit CTRL IgG (C15410206; Diagenode). After elution, qPCRs were performed with a LightCycler 480 (Roche) using a mix of 1× LightCycler 480 SYBR Green I Master (Roche) and 0.5 μM primers (Table S1). For each condition (shCTRL or shAgo1.a), 10% of total chromatin was retained as input CTRL. The efficiency of qPCR amplification was verified and confirmed to be comparable across loci. ChIP enrichment was calculated as percentage of input. To account for potential variation in nucleosome occupancy, enrichment values for H3K9me3 were further normalized to total H3 by dividing each ChIP signal by the corresponding pan-H3 enrichment at the same locus and in the same condition. For comparative analysis, normalized values were then expressed relative to the shCTRL condition, which was set to 100% for each replicate and each locus. This allowed interreplicate comparison of fold changes in histone mark occupancy between shCTRL and shAgo1 conditions.

### smRNA FISH coupled with IF

The smRNA FISH protocol was conducted with the EGFP probes (37). Briefly, the Jurkat polyclonal latent HIV-1 EGFP cells were immobilized on poly-l-lysine (P4707-50ML; Sigma–Aldrich)-coated coverslips in a 24-well plate. Cells were then fixed with 5% paraformaldehyde in PBS for 10 min. Fixed cells were stored in 70% EtOH at 4  °C for a minimum of 1 h to permeabilize the cell membranes. Probes were diluted to a final concentration of 50 nM in 1 g/ml dextran sulfate, 2x side scatter, and 10% formamide and allowed to hybridize at 37  °C overnight in a dark and humid chamber. Wash steps and 4′,6-diamidino-2-phenylindole staining were performed as described (https://www.biosearchtech.com/support/education/stellaris-rna-fish). Then, cells were permeabilized once more with PBS–Triton 0.1% for 10 min at room temperature for Ago1 IF staining. After PBS-mediated washes, a 1/500 anti-Ago1 (Clone 2A7;WAKO) dilution in IF buffer (PBS–Tween 0.05%–bovine serum albumin 0.2%) was incubated on the cells for 1 h at room temperature in a dark chamber. The cells were then washed three times with PBS and Donkey anti-Rabbit Alexa 555 (A-31572; ThermoFisher) was added at a dilution of 1/1000 in IF buffer for 1 h at room temperature in a dark chamber. After three PBS-mediated washes, the coverslips were mounted on slides with the use of ProLong Diamond Antifade Mountant (P36970; ThermoFisher). Imaging was performed with a Spinning-Disk IXplore Olympus confocal microscope, using a 100× objective, a Hamamatsu sCMOS Orca flash 4.0 V3 camera, and the cellSens Dimension acquisition software available at the IMAG’IC core facility. GFP-positive cells were randomly captured.

## Data availability

The data are included in the article and also available upon request.

## Supporting information

This article contains supporting information.

## Conflict of interest

The authors declare that they have no conflicts of interest with the contents of this article.
