# Supplementary material for: Argonaute 1 contributes to the transcriptional silencing of HIV-1
Source: J Biol Chem. 2025 Aug 19;301(10):110612. doi: 10.1016/j.jbc.2025.110612 (PMC12466236; doi:10.1016/j.jbc.2025.110612)
Supplement: Figure S1 [file mmc1.pdf]

A

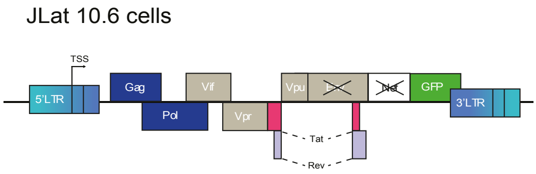

B

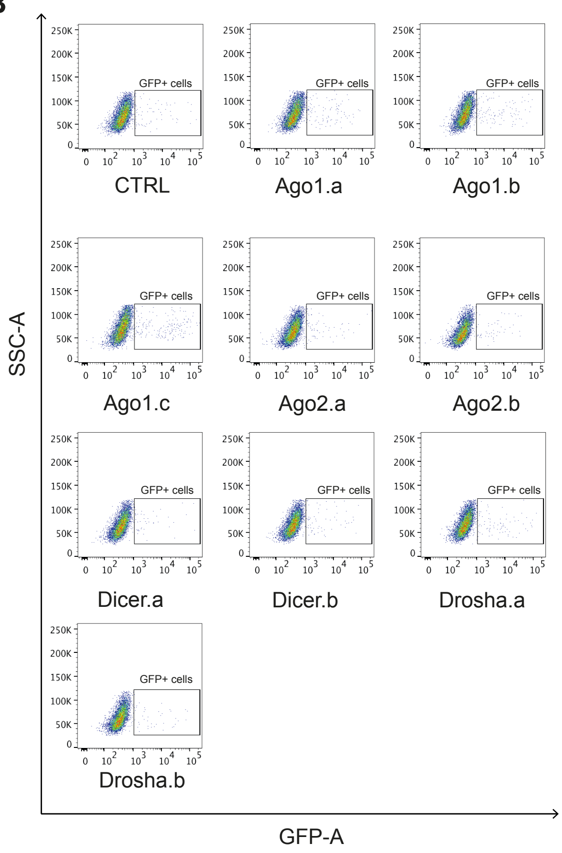

C

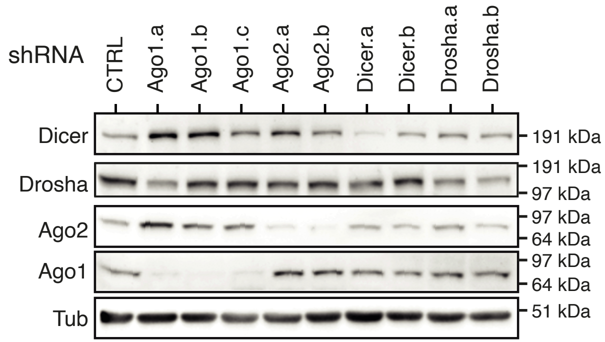

D

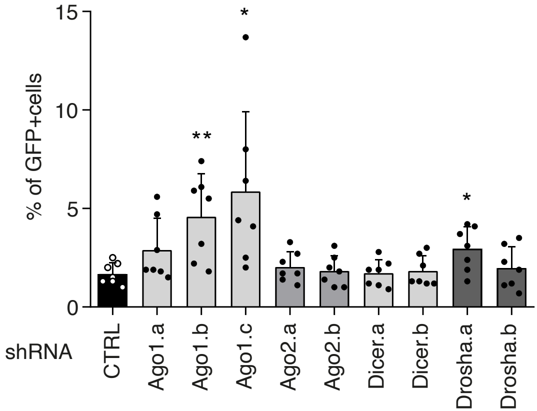

Figure S1: Argonaute 1 downregulation enhances HIV-1 expression in J-Lat 10.6 model of viral latency
(A) Schematic representation of the J-Lat 10.6 genome. **(B)** Flow cytometry analysis of GFP+ cells following transduction of J-Lat 10.6 cells with the indicated shRNAs. A representative experiment is shown. **(C)** Knockdown efficiency in shRNA-transduced J-Lat 10.6 cells was monitored by immunoblotting using the indicated antibodies (representative image of n=7 independent experiments). **(D)** Proviral expression, assessed by the percentage of GFP+ J-Lat 10.6 cells after transduction with shRNA CTRL or shRNAs targeting components of the miRNA pathway. Data represent mean ± SD (n=7 independent experiments). P values were calculated using unpaired t-test (*, p<0.05; **, p<0.01).
