# Supplementary material for: Argonaute 1 contributes to the transcriptional silencing of HIV-1
Source: J Biol Chem. 2025 Aug 19;301(10):110612. doi: 10.1016/j.jbc.2025.110612 (PMC12466236; doi:10.1016/j.jbc.2025.110612)
Supplement: Tables S1-S3 [file mmc2.pdf]

Table S1. Sense oligonucleotide sequences used for shRNA cloning into the pLKO.1 vector

Target gene	Name	Sequence (5' to 3')
Non-targeting	shCtl	CCGGCCTAAGGTTAAGTCGCCCTCGCTCGAGCGAGGGCGACTTAACCTTACGTTTTTG
AGO1	shAgo1.a	CCGGGCTGACAAGAATGAGCGAATTCTCGAGAATTCGCTCATTCTTGTCAGCTTTTTG
	shAgo1.b	CCGGATCAAGCTCCTGGCCAATTACCTCGAGGTAATTGGCCAGGAGCTTGATTTTTTG
	shAgo1.c	CCGGCTGGAGTTACTTTCATAGCATCTCGAGATGCTATGAAAGTAACTCCAGTTTTTG
AGO2	shAgo2.a	CCGGCGGCAAGAAGAGATTAGCAAACTCGAGTTTGCTAATCTCTTCTTGCCGTTTTTG
	shAgo2.b	CCGGACAGATTCCCAAAGGGTAAAGCTCGAGCTTTACCCTTTGGGAATCTGTTTTTTG
DICER	shDicer.a	CCGGGCTCGAAATCTTACGCAAATACTCGAGTATTTGCGTAAGATTTCGAGCTTTTTG
	shDicer.b	CCGGCGGGAGAATTTCAACAGCCAACTCGAGTTGGCTGTTGAAATTCTCCCGTTTTTG
DROSHA	shDrosha.a	CCGGGAGTATTTACTTGCTCAGTAACTCGAGTTACTGAGCAAGTAAATACTCTTTTTG
	shDrosha.b	CCGGCGAAGCTCTTTGGTGAATAATCTCGAGATTATTCACCAAAGAGCTTCGTTTTTG

Table S2. siRNAs used in this study

Target gene	Reference<br>(ON-TARGETplus Horizon Discovery)
AGO1	L-004638-00-0005
CTL	D-001810-10-50

Table S3. Oligonucleotide sequences used for qPCR in this study

**Oligonucleotides sequences**		
**Primers**	**Forward (5' to 3')**	**Reverse (5' to 3')**
RTqPCR		
**GAPDH**	TGCACCACCAACTGCTTAGC	GCATGGACTGTGGTCATGAG
**GFP**	GCATCGACTTCAAGGAGGAC	ACCTTGATGCCGTTCTTCTG
**Luc**	CCTCTAGAGGATGGAACCG	CGCGTACGTGATGTTCACC

**7SK**	CCCTGCTAGAACCTCCAAAC	AAGAAAGGCAGACTGCCAC
**PIG-B**	CCAAGCACTTCTGTCTGCTG	AACACCCATCTTGCCACTTC
**KDSR**	AGATGAGTTGGACCCATTG	AAGCCATGAGTTTCCACCAG
ChIPqPCR		
Nuc-0	ATCTACCACACACAAGGCTA	GTACTAACTTGAAGCACCATCC
GFP	GGTGTTCTGCTGGTAGTGGT	TCGAGGGCGACACCCTG
